# Long-term neurological outcome following out-of-hospital cardiac arrest in Switzerland: a single-centre observational study

**DOI:** 10.3389/fmed.2025.1716369

**Published:** 2026-01-02

**Authors:** Federico Ebert, Mirjam Abt, Tobias Fehr, Katharina Seidl, Markus Huber, Roger Ludwig, Manuela Iten, Robert Greif, Sabine Nabecker, Alexander Fuchs

**Affiliations:** 1 Department of Anaesthesiology and Pain Medicine, Inselspital, Bern University Hospital, University of Bern, Bern, Switzerland; 2 Department of Internal Medicine, Kantonsspital Luzern, Luzern, Switzerland; 3 Department of Intensive Care Medicine, Lindenhofspital, Bern, Switzerland; 4 Schutz & Rettung Bern (SRB), Bern, Switzerland; 5 Department of Intensive Care Medicine, Inselspital, Bern University Hospital, University of Bern, Bern, Switzerland; 6 Faculty of Medicine, University of Bern, Bern, Switzerland; 7 Department of Anesthesiology and Pain Management, Sinai Health System, University of Toronto, Toronto, ON, Canada

**Keywords:** ECPR, neurological outcome, OHCA, out-of-hospital cardiac arrest, survival

## Abstract

**Background:**

Favourable neurological outcome in patients with out-of-hospital cardiac arrest (OHCA) vary across countries. Different advanced resuscitation strategies such as extracorporeal cardiopulmonary resuscitation (ECPR) might have impact on long-term neurological outcome. However, this remains unclear in Switzerland.

**Methods:**

This retrospective single-centre observational study included all patients with OHCA transported by the local emergency medical services to a large Swiss academic hospital between 1 January 2015 and 31 December 2023. Data were collected before and after the implementation of the local ECPR programme for patients with refractory OHCA on 01 May 2018. The primary outcome was 1-year favourable neurological outcome, defined as Cerebral Performance Categories 1 and 2. Secondary outcomes included 30-day favourable neurological outcome, characteristics and survival of patients treated with ECPR, and factors associated with non-survival among all OHCA patients.

**Results:**

A total of 578 patients with OHCA were transported to the hospital. Favourable neurological survival at 1 year was 16.8% (95%-CI, 12.1–22.4%) before and 21.5% (95%-CI, 17.4–26.1%) after the ECPR programme implementation. Hazard ratios for overall survival were 2.19 for patients with a non-shockable initial rhythm, 1.02 for older age and 1.68 for unwitnessed OHCA. Of all transported patients, 16.8% (*n* = 97, *n* = 31 before vs. *n* = 66 after) met local ECPR criteria. In total 34 patients with refractory OHCA were treated with ECPR, all assessable survivors had favourable 1-year neurological outcomes.

**Conclusion:**

This observational study on patients sustaining OHCA transferred to a large Swiss hospital showed 1 year favourable outcome in 19.7% (95%-CI: 16.6–23.2%). Among ECPR patients, all five survivors had a favourable neurological outcome at 1 year. No association was found between implementing an ECPR programme for patients with refractory OHCA and 1 year favourable neurological outcome. However, the effect might be underestimated given the low incidence of ECPR.

**Clinical trial registration:**

https://www.clinicaltrials.gov, identifier NCT03759210.

## Introduction

Sudden cardiac arrest is the third leading cause of death in Europe (1). In Switzerland, out-of-hospital cardiac arrest (OHCA) accounts for approximately 8,000 deaths annually (2). The incidence of cardiac arrest in Switzerland is 78 cases per 100,000 person-years (3). Reported survival rates for OHCA in adult patients range from 8 to 10% (4, 5). Despite advancements in resuscitation science, overall survival after cardiac arrest remains low, and substantial survivors suffer from neurological impairment, particularly following refractory cardiac arrest (6).

Extracorporeal cardiopulmonary resuscitation (ECPR) is an emerging advanced resuscitation technique with the aim of improving survival following cardiac arrest. In addition, ECPR may offer the chance to preserve organs for transplantation in selected cases. The International Liaison Committee on Resuscitation (ILCOR) and international resuscitation guidelines suggest ECPR as a rescue therapy for selected patients with refractory cardiac arrest (7, 8). The use of ECPR has increased more than tenfold over the past 20 years (9). A recent meta-analysis found that ECPR reduces in-hospital mortality and improves survival up to 30-day survival for patients sustaining OHCA. Furthermore, ECPR was associated with improved neurological outcome in OHCA survivors at short-term follow-up to 30 days (10). A systematic review suggests that ECPR might improve survival and neurological outcomes in patients with refractory OHCA (11). However, careful patient selection appears to play an important role in the beneficial effects of ECPR (11).

In contrast, several studies have reported no beneficial effects of ECPR on overall hospital survival and neurological outcome (12, 13). Little is known about the impact of ECPR on survival in patients with refractory OHCA in Switzerland. Especially long-term favourable neurological outcome in OHCA survivors remains unclear. The Bern University Hospital implemented ECPR as a standard treatment programme for patients sustaining refractory OHCA in May 2018.

The aim of this study was to investigate long-term neurological outcomes of patients with OHCA transferred to a large Swiss University Hospital. Furthermore, we investigated the association of the implementation of an ECPR programme for patients with refractory OHCA. Our data might contribute to the broader understanding of the impact of OHCA on long-term neurological outcomes.

## Materials and methods

### Study design

This retrospective observational cohort study was approved by the Cantonal Ethics Committee of Bern (Project ID 2019-01628, 03/09/2019, Prof. C. Seiler) and conducted in accordance with the Declaration of Helsinki. The reporting adhered to the applicable Strengthening the Reporting of Observational Studies in Epidemiology (STROBE) guidelines (14).

### Setting

The study was conducted at the University Hospital of Bern, Switzerland and the Bern Emergency Medical Service (EMS) Schutz und Rettung Bern. The EMS covers the city of Bern and its surroundings, serving around 430,000 inhabitants and handling approximately 20,000 primary rescue missions per year. We included patients with OHCA from the EMS database from 01 January 2015 to 31 December 2023. On 01 May 2018, the University Hospital Bern implemented a 24 h/7d ECPR programme in collaboration with the Bern EMS for patients suffering from refractory OHCA. Patients were eligible for ECPR with the following criteria:

- Age: ≤ 70 years, in good physical health
- First initial rhythm: ventricular fibrillation or ventricular tachycardia
- Downtime ≤ 5 min
- Initial and current EtCO_2_ ≥ 15 mmHg
- Sufficient lay person resuscitation ≤ 15 min
- Arrival at the emergency department within 45 min after out-of-hospital cardiac arrest under ongoing chest compressions

Patients were excluded from ECPR if any of the following criteria applied:

- A living will exclude resuscitation measures
- Severe trauma
- Severe systemic disease with limited prognosis (cirrhosis of the liver > Child A, COPD > Gold III, tumour disease without curative healing potential, polymorbidity)

All patients were treated according to European Resuscitation Council (ERC) Advanced Life Support (ALS) guidelines (8). After 10 min of refractory OHCA, potential candidates for ECPR were evaluated on-scene by the treating EMS physician. If the inclusion criteria were met, the patient was transported by ambulance to the Emergency Department of the University Hospital, continuing with ALS, including mechanical chest compression (LUCAS 2, Jolife AB, Lund, Sweden). Upon arrival at the emergency department, an independent multidisciplinary team, uninvolved in the preceding prehospital management, conducted a brief re-evaluation of the patient to determine eligibility for ECPR. In case ECPR was not indicated, standard ALS was continued by the Emergency Department team. In case ECPR was indicated, cannulation was performed under ongoing chest compression by the interventional cardiologists in the catheter lab. In the rare case that an interventional suite was not available, cannulation was performed by a trained intensivist in the intensive care unit (ICU). The extracorporeal perfusion, further diagnostic procedures and treatments were performed in the ICU. In the case of return of spontaneous circulation (ROSC) before hospital arrival, the patient underwent further diagnostic procedures in the Emergency Department (e.g., computer tomography) and was subsequently treated in the ICU.

Prior to the implementation of the local ECPR programme, patients were treated according to ERC guidelines and transported to the hospital only if ROSC was achieved or if ongoing resuscitation was warranted due to suspected coronary thrombosis or pulmonary embolism. Before May 2018, ECPR was performed in a limited number of cases in the ICU, but not as part of a standardised local programme. During the unprecedented COVID-19 pandemic, the ECPR programme was partially suspended due to intensive care unit capacities between April and June 2020, and again from November 2020 to January 2021. This led us to prolong the phase after the ECPR programme implementation.

### Participants and data source

We screened the EMS database for eligible patients with a National Advisory Committee for Aeronautics Score ≥ 5. We included all patients with OHCA who were treated with ALS by the EMS and transported to the Emergency Department. We excluded patients declared dead on-scene with or without resuscitation attempt.

### Variables

We recorded the patient’s demographics (age, sex, co-morbidities), cardiac arrest-related data (witnessed cardiac arrest, layperson CPR, first rhythm, event location), and pre-hospital times (EMS Respond time: interval from the emergency call to the arrival of EMS at the patient, EMS Pre-hospital time: duration from the emergency call to EMS arrival at the emergency department).

In-hospital data included ECPR and survival data (i.e., emergency department, ICU, hospital discharge). We recorded survival after 30 days, hospital discharge, 1 year and survival status at the end of the study period. Furthermore we assessed the Cerebral Performance Category (CPC) (15) at hospital discharge, after 30 days and 1 year. All data were recorded in a departmental REDCap server (REDCap, Vanderbilt University, Pennsylvania, USA).

### Outcomes

The primary outcome was favourable neurological outcome 1 year after OHCA, defined by a CPC score of 1 or 2. Secondary outcomes included survival to hospital discharge, 30-day, and overall survival, and CPC at 30-day. Data were collected from EMS and hospital medical records. Follow-up was performed through telephone interviews with the patient or their general practitioner by a person not involved in the clinical care and blinded to the type of treatment.

### Statistical analysis and sample size

Due to the retrospective and explorative character of the study, we did not calculate a formal sample size.

Categorical variables were given in numbers and percentages. Continuous variables were given as means and standard deviation (SD), and skewed data with median and first and third quartiles [Q1; Q3]. With respect to group comparisons, Student’s *t*-tests were used to compare continuous, normally distributed data, and Mann–Whitney or Kruskal–Wallis tests for skewed data. Categorical variables were compared with chi-squared tests or Fisher’s exact tests.

A survival analysis using Kaplan–Meier estimates was computed for the entire cohort stratified according to period (prior to or after introduction of ECPR programme on 1st May 2018). Additionally, a cox regression model was computed with period (as above), age, sex, prehospital time, rhythm and an indicator if the cardiac arrest was witnessed as covariates.

We did not impute missing data.

The significance level of probability was defined as ≤0.05. Given the exploratory character of the analysis, no *p*-value adjustments for multiple comparisons were performed. All calculations were performed with R statistical software.

## Results

**Figure 1:**
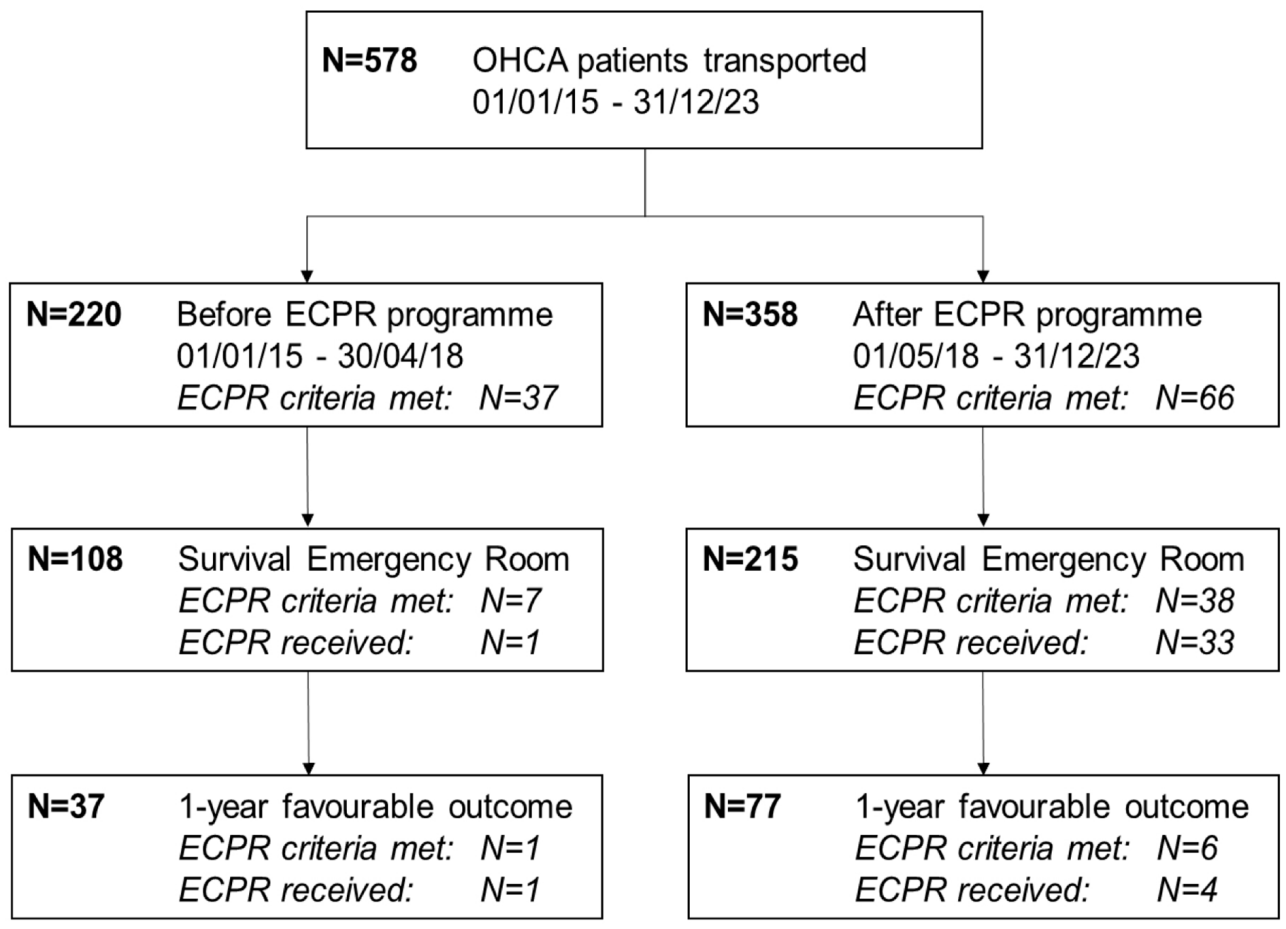
Study flow chart. OHCA, out-of-hospital cardiac arrest; ECPR, extracorporeal cardiopulmonary resuscitation.

We screened 1,889 patients with a primary EMS mission for a patient with OHCA; of these, 578 were transported to the hospital and included in this analysis (Figure 1).

The median age of the included OHCA patients transported to the hospital was 65.7 [54.1; 76.5] years, 24.6% were female, and 42.6% had an initial shockable rhythm (Table 1). Following the implementation of the ECPR programme, the median pre-hospital EMS time significantly decreased from 55 [43.0; 71.0] minutes to 47 [37.0, 58.0] minutes (*p* = 0.001).

**Table 1:** Baseline characteristics of all patients sustaining out-of-hospital cardiac arrest transported to the hospital before and after extracorporeal cardiopulmonary resuscitation (ECPR) programme implementation.

Variables	All	Before ECPR programme	After ECPR programme	*p*	*N*
		(01/2015–04/2018)	(05/2018–12/2023)		
	*N* = 578	*N* = 220	*N* = 358		
Age at event (years)	65.7 [54.1; 76.5]	65.9 [53.8; 76.1]	65.6 [54.3; 76.8]	0.937	578
Sex (female)	142 (24.6%)	50 (22.8%)	92 (25.7%)	0.499	577
Event location				0.104	576
At patient home	251 (43.6%)	93 (42.7%)	158 (44.1%)		
In public	263 (45.7%)	108 (49.5%)	155 (43.3%)		
Nursing home	12 (2.1%)	4 (1.8%)	8 (2.2%)		
Psychiatric facility	11 (1.9%)	2 (0.9%)	9 (2.5%)		
Not documented	2 (0.3%)	2 (0.9%)	0 (0.0%)		
Other	37 (6.4%)	9 (4.1%)	28 (7.8%)		
Witnessed cardiac arrest				0.047	577
Yes	398 (69.0%)	142 (64.8%)	256 (71.5%)		
No	171 (29.6%)	76 (34.7%)	95 (26.5%)		
Not documented	8 (1.4%)	1 (0.5%)	7 (2.0%)		
Bystander CPR				0.971	576
Yes	257 (44.6%)	97 (44.3%)	160 (44.8%)		
No	319 (55.4%)	122 (55.7%)	197 (55.2%)		
Use of AED				0.238	577
Yes	115 (19.9%)	36 (16.4%)	79 (22.1%)		
No	428 (74.2%)	171 (77.7%)	257 (72.0%)		
Unknown	34 (5.9%)	13 (5.9%)	21 (5.9%)		
AED shock				0.585	115
Yes	84 (73.0%)	28 (77.8%)	56 (70.9%)		
No	31 (27.0%)	8 (22.2%)	23 (29.1%)		
Initial rhythm documented by EMS					578
VF	230 (39.8%)	83 (37.7%)	147 (41.1%)		
Pulsless VT	12 (2.1%)	1 (0.5%)	11 (3.1%)		
Shockable (AED) but not specified	4 (0.7%)	1 (0.5%)	3 (0.8%)		
PEA	175 (30.3%)	68 (30.9%)	107 (29.9%)		
Asystole	121 (20.9%)	51 (23.2%)	70 (19.6%)		
Non-shockable but not specified	16 (2.8%)	6 (2.7%)	10 (2.8%)		
Unknown	10 (1.7%)	2 (0.9%)	8 (2.2%)		
Other	10 (1.7%)	8 (3.6%)	2 (0.6%)		
EtCO_2_ on arrival at the emergency department (mmHg)	35.0 [25.0; 41.0]	35.0 [25.0; 40.8]	34.0 [25.0; 41.0]	0.425	430
EMS respond time (Call until EMS arrives at location) (min)	10.0 [8.0; 13.0]	10.0 [8.0; 14.0]	10.0 [7.0; 13.0]	0.066	577
EMS pre-hospital time (Call until EMS arrives at emergency department) (min)	50.0 [40.0; 61.8]	55.0 [43.0; 71.0]	47.0 [37.0; 58.0]	<0.001	574

### Primary outcome

Of the 578 OHCA patients transported to the hospital during the study period, 19.7% (95%-CI: 16.6–23.2%) had a favourable neurological outcome 1 year after OHCA. Before the implementation of the ECPR programme, it was 16.8% (95%-CI: 12.1–22.4%), and after 21.5% (95%-CI: 17.4–26.1%) (*p* = 0.356) (Table 2). The adjusted odds ratios comparing the period before and after ECPR programme implementation are given in Table 3.

**Table 2:** Return of spontaneous circulation (ROSC), survival and neurological outcome for all patients transported to the hospital before and after the implementation of the extracorporeal cardiopulmonary resuscitation (ECPR) programme.

ROSC, Survival and CPC	All patients	Before ECPR programme	After ECPR programme	*p*	*N*
	*N* = 578	*N* = 220	*N* = 358		
ROSC					
On-scene	357 (61.8, 95%-CI: 57.7–65.7%)	125 (56.8, 95%-CI: 50.0–63.5%)	232 (64.8, 95%-CI: 59.6–69.8%)	0.067	578
Duration of CPR until ROSC, min	16.0 [8.0; 26.0]	20.0 [10.0; 30.0]	15.0 [6.0; 25.0]	0.015	282
Hospital arrival	293 (50.8, 95%-CI: 46.6–54.9%)	111 (50.5, 95%-CI: 43.7–57.2%)	182 (51.0, 95%-CI: 45.7–56.3%)	0.970	577
ECPR started	34 (5.9, 95%-CI: 4.1–8.1%)	4 (1.8, 95%-CI: 0.5–4.6%)	30 (8.4, 95%-CI: 5.7–11.7%)	0.002	577
Condition after ECPR				>0.99	34
Alive	10 (29.4, 95%-CI: 15.1–47.5%)	1 (25.0, 95%-CI: 0.6–80.6%)	9 (30.0, 95%-CI: 14.7–49.4%)		
Dead	24 (70.6, 95%-CI: 52.5–84.9%)	3 (75.0, 95%-CI: 19.4–99.4%)	21 (70.0, 95%-CI: 50.6–85.3%)		
Survival					
Emergency department	323 (55.9, 95%-CI: 51.7–60.0%)	108 (49.1, 95%-CI: 42.3–55.9%)	215 (60.1, 95%-CI: 54.8–65.2%)	0.013	578
Intensive care unit	162 (28.0, 95%-CI: 24.4–31.9%)	59 (26.8, 95%-CI: 21.1–33.2%)	103 (28.8, 95%-CI: 24.1–33.8%)	0.680	578
Hospital discharge	158 (27.3, 95%-CI: 23.7–31.2%)	53 (24.1, 95%-CI: 18.6–30.3%)	105 (29.3, 95%-CI: 24.7–34.3%)	0.202	578
30 days	143 (25.0, 95%-CI: 21.5 28.7%)	47 (21.8, 95%-CI: 16.4–27.9%)	96 (26.9, 95%-CI: 22.4–31.8%)	0.202	573
1 year	134 (23.6, 95%-CI: 20.1–27.3%)	43 (20.1, 95%-CI: 14.9–26.1%)	91 (25.6, 95%-CI: 21.2–30.5%)	0.159	569
Discharged to				0.389	158
Home	69 (43.7, 95%-CI: 35.8–51.8%)	26 (49.1, 95%-CI: 35.1–63.2%)	43 (41.0, 95%-CI: 31.5–51.0%)		
Nursing home/retirement home	2 (1.3, 95%-CI: 0.2–4.5%)	1 (1.9, 95%-CI: <0.1–10.1%)	1 (1.0, 95%-CI: <0.1–5.2%)		
Other hospital	32 (20.3, 95%-CI: 14.3–27.4%)	12 (22.6, 95%-CI: 12.3–36.2%)	20 (19.0, 95%-CI: 12.0–27.9%)		
Rehabilitation facility	55 (34.8, 95%-CI: 27.4–42.8%)	14 (26.4, 95%-CI: 15.3–40.3%)	41 (39.0, 95%-CI: 29.7–49.1%)		
Patients who were uncontrolled organ donor	2 (8.3, 95%-CI: 1.0–27.0%)	0 (0.0, 95%-CI: 0.0–70.8%)	2 (9.5, 95%-CI: 1.2–30.4%)	>0.99	24
Cerebral performance category (CPC)					
Hospital discharge					
*Categories*				0.273	578
CPC 1	106 (18.3, 95%-CI: 15.3–21.7%)	34 (15.5, 95%-CI: 10.9–20.9%)	72 (20.1, 95%-CI: 16.1–24.6%)		
CPC 2	36 (6.2, 95%-CI: 4.4–8.5%)	13 (5.9, 95%-CI: 3.2–9.9%)	23 (6.4, 95%-CI: 4.1–9.5%)		
CPC 3	12 (2.1, 95%-CI: 1.1–3.6%)	6 (2.7, 95%-CI: 1.0–5.8%)	6 (1.7, 95%-CI: 0.6–3.6%)		
CPC 4	4 (0.7, 95%-CI: 0.2–1.8%)	0 (0.0, 95%-CI: 0.0–1.7%)	4 (1.1, 95%-CI: 0.3–2.8%)		
CPC 5	420 (72.7, 95%-CI: 68.8–76.3%)	167 (75.9, 95%-CI: 69.7–81.4%)	253 (70.7, 95%-CI: 65.7–75.3%)		
Unknown	0 (0.0, 95%-CI: 0.0–0.6%)	0 (0.0, 95%-CI: 0.0–1.7%)	0 (0.0, 95%-CI: 0.0–1.0%)		
*Favourable outcome*				0.193	578
30 days					
*Categories*				0.541	578
CPC 1	103 (17.8, 95%-CI: 14.8–21.2%)	32 (14.5, 95%-CI: 10.2–19.9%)	71 (19.8, 95%-CI: 15.8–24.3%)		
CPC 2	22 (3.8, 95%-CI: 2.4–5.7%)	8 (3.6, 95%-CI: 1.6–7.0%)	14 (3.9, 95%-CI: 2.2–6.5%)		
CPC 3	4 (0.7, 95%-CI: 0.2–1.8%)	2 (0.9, 95%-CI: 0.1–3.2%)	2 (0.6, 95%-CI: 0.1–2.0%)		
CPC 4	0 (0.0, 95%-CI: 0.0–0.6%)	0 (0.0, 95%-CI: 0.0–1.7%)	0 (0.0, 95%-CI: 0.0–1.0%)		
CPC 5	435 (75.3, 95%-CI: 71.5–78.7%)	173 (78.6, 95%-CI: 72.6–83.9%)	262 (73.2, 95%-CI: 68.3–77.7%)		
Unknown	14 (2.4, 95%-CI: 1.3–4.0%)	5 (2.3, 95%-CI: 0.7–5.2%)	9 (2.5, 95%-CI: 1.2–4.7%)		
*Favourable outcome*				0.275	578
CPC 1 or 2	125 (21.6, 95%-CI: 18.3–25.2%)	40 (18.2, 95%-CI: 13.3–23.9%)	85 (23.7, 95%-CI: 19.4–28.5%)		
1 year					
*Categories*				0.428	578
CPC 1	102 (17.6, 95%-CI: 14.6–21.0%)	34 (15.5, 95%-CI: 10.9–20.9%)	68 (19.0, 95%-CI: 15.1–23.4%)		
CPC 2	12 (2.1, 95%-CI: 1.1–3.6%)	3 (1.4, 95%-CI: 0.3–3.9%)	9 (2.5, 95%-CI: 1.2–4.7%)		
CPC 3	4 (0.7, 95%-CI: 0.2–1.8%)	2 (0.9, 95%-CI: 0.1–3.2%)	2 (0.6, 95%-CI: 0.1–2.0%)		
CPC 4	0 (0.0, 95%-CI: 0.0–0.6%)	0 (0.0, 95%-CI: 0.0–1.7%)	0 (0.0, 95%-CI: 0.0–1.0%)		
CPC 5	444 (76.8, 95%-CI: 73.2–80.2%)	177 (80.5, 95%-CI: 74.6–85.5%)	267 (74.6, 95%-CI: 69.7–79.0%)		
Unknown	16 (2.8, 95%-CI: 1.6–4.5%)	4 (1.8, 95%-CI: 0.5–4.6%)	12 (3.4, 95%-CI: 1.7–5.8%)		
*Favourable outcome*				0.186	578
CPC 1 or 2	114 (19.7, 95%-CI: 16.6–23.2%)	37 (16.8, 95%-CI: 12.1–22.4%)	77 (21.5, 95%-CI: 17.4–26.1%)		

Data are given in *n* (%, 95 confidence interval) or median [Q1; Q3].

**Table 3:** Adjusted odds ratio (OR) with comparison of the period before and after the ECPR programme implementation.

Characteristic	OR	95% CI	*P*
CPC at 1-year			
CPC 1–2	–	–	
CPC 3–5	0.8	0.48, 1.32	0.4
CPC unknown	1.68	0.46, 8.00	0.5
Age (years)	1	0.99, 1.01	0.8
Sex			
Male	–	–	
Female	1.14	0.76, 1.73	0.5
Witnessed cardiac arrest			
Yes	–	–	
No	0.71	0.48, 1.04	0.078
Not documented	3.86	0.67, 72.8	0.2
Initial rhythm			
Non-shockable	–	–	
Shockable	1.21	0.83, 1.76	0.3

CI, Confidence Interval; OR, Odds Ratio.

### Secondary outcomes

Data on ROSC, survival, and neurological outcomes are in Table 2. The duration of CPR to ROSC was 20 [10.0; 30.0] minutes before and 15 [6.0; 25.0] minutes after the implementation of the ECPR programme (*p* = 0.015). ECPR was performed in 1.8% (95% CI: 0.5%; 4.6%) of OHCA patients before and 8.4% (95% CI: 5.7%; 11.7%) after the implementation of the ECPR programme (*p* = 0.002). Furthermore, following the implementation of the programme, there was an increase in patient survival rates in the emergency department, with 49.1% (95% CI: 42.3–55.9%) surviving initially and 60.1% (95% CI: 54.8–65.2%) surviving thereafter (*p* = 0.013). Of the 578 OHCA patients transported to the hospital during the study period, 21.6% (95% CI: 18.3–25.2%) had a favourable neurological outcome 30 days after OHCA. Before the implementation of the ECPR programme, it was 18.2% (95% CI: 13.3–23.9%), and after 23.7% (95% CI: 19.4–28.5%), without statistical significance (*p* = 0.218) (Table 2).

**Figure 2:**
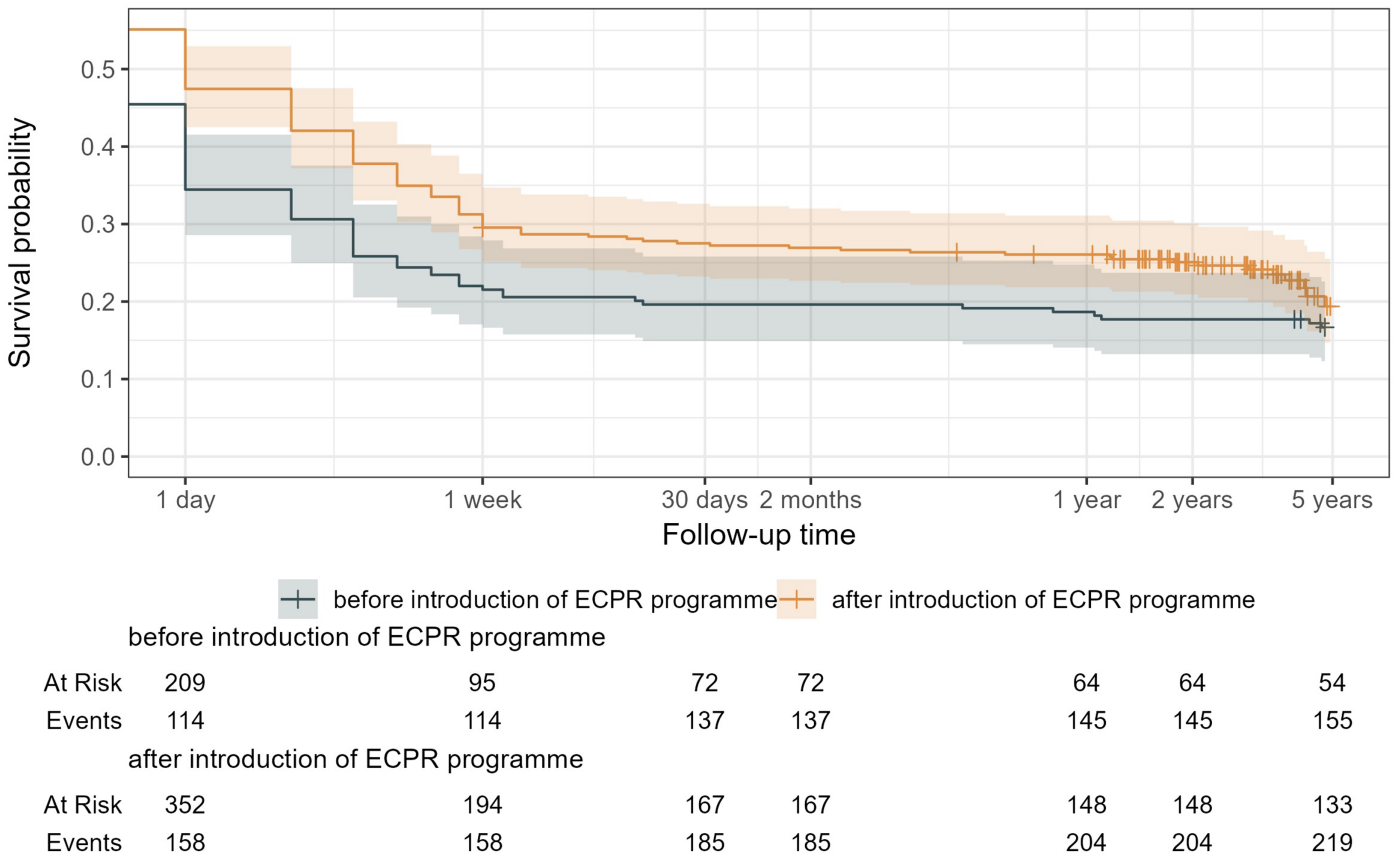
Kaplan Meier with overall long-term survival probability before (grey) and after (orange) implementation of the ECPR programme.

Figure 2 shows the Kaplan Meier survival analysis (Figure 2). Following the implementation of the ECPR programme, there was an unadjusted improvement in overall survival (*p* = 0.041).

Table 4 presents the Cox regression analysis for the overall survival for patients transported to the hospital after OHCA (Table 4). Older age (*p* < 0.001), unwitnessed cardiac arrest (*p* < 0.001) and an initial non-shockable rhythm (*p* < 0.001) were independently associated with decreased overall survival in patients with OHCA (Table 4).

**Table 4:** Hazard ratio (HR) COX regression for overall survival over the study period for all out-of-hospital cardiac arrest (OHCA) patients transported to the hospital.

Characteristic	HR (1)	95% CI	*p*-value
Period			
Before ECPR programme	–	–	
After ECPR programme	0.88	0.72, 1.07	0.2
Age (years)	1.02	1.01, 1.02	<0.001
Sex			
Male	–	–	
Female	0.79	0.63, 0.99	0.052
Witnessed cardiac arrest			
Yes	–	–	
No	1.68	1.36, 2.08	<0.001
Rhythm			
Shockable	–	–	
Non-shockable	2.19	1.78, 2.69	<0.001
Prehospital time (min)	1	1.00, 1.00	>0.9

ECPR, extracorporeal cardiopulmonary resuscitation.

### Patients with refractory out-of-hospital cardiac arrest

Of the 578 OHCA patients transported to the hospital during the study period, 97 (*n* = 31 before vs. *n* = 66 after ECPR programme implementation) were admitted to the ER with refractory cardiac arrest under ongoing mechanical chest compressions. The adjusted odds ratio for survival of the emergency department was 5.0 (95%-CI: 1.9–15.1, *p* = 0.002) (Table 5). The adjusted odds ratio for favourable neurological outcome at 1 year after OHCA was 3.1 (95%-CI: 0.5–60.0, *p* = 0.3). Similarly, favourable neurological outcome at 30-days did not improve.

**Table 5:** Survival and neurological outcomes for patients with refractory out-of-hospital cardiac arrest fulfilling local ECPR criteria stratified for the period before and after ECPR programme implementation.

Survival and neurological outcome	Before ECPR	After ECPR	*P*	Adjusted odds ratio^†^
	*N* = 31	*N* = 66		After ECPR vs. Before ECPR
Survival				
Emergency department	7 (22.6%)	38 (57.6%)	0.003	5.0 (95%-CI: 1.9–15.1, *P* = 0.002)
ICU	3 (9.7%)	7 (10.6%)	>0.99	1.3 (95%-CI: 0.3–6.7, *p* = 0.7)
Hospital discharge	3 (9.7%)	7 (10.6%)	>0.99	1.3 (95%-CI: 0.3–6.7, *p* = 0.7)
30 days	2 (6.4%)	6 (9.1%)	>0.99	1.4 (95%-CI: 0.3–10.3, *p* = 0.7)
1 year	2 (6.4%)	6 (9.1%)	>0.99	1.4 (95%-CI: 0.3–10.3, *p* = 0.7)
Favourable neurological outcome: CPC I or II				
Hospital discharge	2 (6.4%)	6 (9.1%)	>0.99	1.6 (95%-CI: 0.3–11.9, *p* = 0.6)
30 days	1 (3.2%)	6 (9.1%)	0.424	3.1 (95%-CI: 0.5–60.0, *p* = 0.3)
1 year	1 (3.2%)	6 (9.1%)	0.424	3.1 (95%-CI: 0.5–60.0, *p* = 0.3)

^†^We adjusted for the variables age, sex, wittnessed cardiac arrest, first rhythm, and prehospital time.

### ECPR cohort

The median age of the 34 patients with refractory OHCA treated with ECPR was 55.7 years [46.7; 60.1], and 11.8% (4 patients) were female (Table 6). While four patients were treated before the ECPR programme was implemented, 30 were treated afterwards. Six patients (17.6%) survived to hospital discharge following ECPR treatment, all with favourable neurological outcomes. One patient was lost to follow-up after returning to their home country. Among the five assessable survivors, all showed a favourable neurological outcome at both the 30-day and 1-year follow-up.

**Table 6:** Baseline characteristics and outcomes specifically for patients treated with ECPR.

Characteristics and outcome	ECPR patients	*N*
	*N* = 34	
ECPR duration (h)	20.1 [10.8; 50.3]	34
Age at event (years)	55.7 [46.7; 60.1]	34
Sex (female)	4 (11.8%)	34
Pre-existing co-morbidity:		34
None	24 (70.6%)	
COPD Gold >II	1 (2.9%)	
Home oxygen therapy	1 (2.9%)	
Polymorbidity	6 (17.6%)	
No data available	3 (8.8%)	
Event location		34
At home	12 (35.3%)	
In public	19 (55.9%)	
Other	3 (8.8%)	
CPR		34
Ongoing at arrival EMS	23 (67.6%)	
Start at arrival EMS	11 (32.4%)	
Witnessed cardiac arrest		34
Yes	27 (79.4%)	
CPR performed by lay person		34
Yes	15 (44.1%)	
No	19 (55.9%)	
AED attached		34
Yes	7 (20.6%)	
No	25 (73.5%)	
Unknown	2 (5.9%)	
AED used		7
Yes	7 (100.0%)	
Total number of shocks		7
1	2 (28.6%)	
2	4 (57.1%)	
3	1 (14.3%)	
Initial rhythm documented by EMS		34
VF	25 (73.5%)	
Pulseless VT	1 (2.9%)	
PEA	7 (20.6%)	
Asystole	1 (2.9%)	
First documented etCO_2_ after airway intervention	35.1 (14.9)	28
Final etCO_2_ on arrival at the emergency department	29.0 [22.0; 37.2]	32
Respond time (Call 144 until EMS arrives at location) (min)	9.2 (3.2)	34
Prehospital time (call 144 until EMS arrives at emergency department) (min)	41.5 [33.0; 48.8]	34
*Instance of ROSC for at least 1 min*		
On-scene		34
Yes	20 (58.8%)	
No	14 (41.2%)	
Hospital arrival		34
Yes	3 (8.8%)	
No	31 (91.2%)	
*Condition after ECPR*		34
Alive	10 (29.4%)	
Dead	24 (70.6%)	
*Survival*		
Emergency department (Yes)	31 (91.2, 95%-CI: 76.3–98.1%)	34
ICU (Yes)	6 (17.6, 95%-CI: 6.8–34.5%)	34
Hospital discharge (Yes)	6 (17.6, 95%-CI: 6.8–34.5%)	34
30 days (Yes)	5 (15.2, 95%-CI: 5.1–31.9%)	33
1 year (Yes)	5 (15.2, 95%-CI: 5.1–31.9%)	33
Discharged to		6
Home	3 (50.0, 95%-CI: 11.8–88.2%)	
Other hospital	2 (33.3, 95%-CI: 4.3–77.7%)	
Rehabilitation facility	1 (16.7, 95%-CI: 0.4–64.1%)	
Patients who were uncontrolled organ donor	2 (8.3, 95%-CI: 1.0–27.0%)	24
*Clinical performance category (CPC)*		
Hospital discharge		34
*Categories*		
CPC 1	4 (11.8, 95%-CI: 3.3–27.5%)	
CPC 2	2 (5.9, 95%-CI: 0.7–19.7%)	
CPC 3	0 (0.0, 95%-CI: 0.0–10.3%)	
CPC 4	0 (0.0, 95%-CI: 0.0–10.3%)	
CPC 5	28 (82.4, 95%-CI: 65.5–93.2%)	
Unknown	0 (0.0, 95%-CI: 0.0–10.3%)	
*Favourable outcome*		
CPC 1 or 2	6 (17.6, 95%-CI: 6.8–34.5%)	
30 days		34
*Categories*		
CPC 1	4 (11.8, 95%-CI: 3.3–27.5%)	
CPC 2	1 (2.9, 95%-CI: 0.1–15.3%)	
CPC 3	0 (0.0, 95%-CI: 0.0–10.3%)	
CPC 4	0 (0.0, 95%-CI: 0.0–10.3%)	
CPC 5	29 (85.3, 95%-CI: 68.9–95.0%)	
Unknown	0 (0.0, 95%-CI: 0.0–10.3%)	
*Favourable outcome*		
CPC 1 or 2	5 (14.7, 95%-CI: 5.0–31.1%)	
1 year		34
*Categories*		
CPC 1	4 (11.8, 95%-CI: 3.3–27.5%)	
CPC 2	1 (2.9, 95%-CI: 0.1–15.3%)	
CPC 3	0 (0.0, 95%-CI: 0.0–10.3%)	
CPC 4	0 (0.0, 95%-CI: 0.0–10.3%)	
CPC 5	29 (85.3, 95%-CI: 68.9–95.0%)	
Unknown	0 (0.0, 95%-CI: 0.0–10.3%)	
*Favourable outcome*		
CPC 1 or 2	5 (14.7, 95%-CI: 5.0–31.1%)	

Data are given in *n* (%) or median [Q1; Q3].

^*^One patient was alive at hospital discharge with neurological favourable survival but lost to 30-day and 1-year follow-up due to transfer to the country of origin.

Among the 28 (82.4%) non-survivors after ECPR, two patients became uncontrolled organ donors (8.4%), resulting in successful organ transplantation.

## Discussion

Long-term favourable neurological outcomes in an OHCA cohort transferred to a large Swiss University hospital improved from 16.8% (95%-CI: 12.1–22.4%) to 21.5% (95%-CI: 17.4–26.1%) over the 8-year study period. Out of 558 patients with OHCA transported to the hospital, 34 patients with refractory OHCA were treated with ECPR. Of these, six had a favourable neurological outcome after 1 year. The implementation of an ECPR programme was not associated with significant changes in favourable neurological outcomes 1 year after the event. Older age, non-witnessed cardiac arrest, and a non-shockable rhythm were associated with lower survival rates after OHCA in this cohort.

### Long-term favourable neurological outcome

Little is known about long-term survival in OHCA patients, especially after the implementation of an ECPR programme. Some studies (16–18) demonstrated a beneficial effect of ECPR on favourable neurological outcomes 6 months after OHCA, but there is a paucity in the reporting of 1-year neurological outcomes. A single-centre randomised controlled trial in the Czech Republic reported improved neurological outcome at 30 days in patients in the ECPR group (17). In the long-term follow-up of this study (19), patients had a median follow-up of approximately 5 years, and the authors did not find a significant difference in neurological outcomes between the two groups. This is consistent with our findings, as the ECPR programme was not associated with a significant effect on neurological outcome at 1-year follow-up.

### Short-term favourable neurological outcome

A recent registry study compared the survival and 30-day neurological outcome before and after the implementation of an ECPR programme for patients with refractory OHCA and presumed cardiac aetiology in an urban setting in Norway (20). In contrast to our result, the proportion of survivors with favourable neurological outcome was lower after the implementation of their ECPR programme (20). However, only 14 patients were treated with ECPR, which may introduce uncertainties regarding the statistical power of the analysis.

Two meta-analyses, including 20 studies, suggest that ECPR following OHCA is associated with short-term favourable neurological outcomes (10, 11). However, short-term neurological outcome might not be the best parameter to evaluate the impact of ECPR as there might be some patients with delayed improvement (21).

### Short-term and long-term favourable neurological outcome in patients with refractory out-of-hospital cardiac arrest eligible for ECPR

Our observations from the overall cohort were transferable to the subgroup fulfilling local ECPR criteria. Although we did not find an association between ECPR and a favourable outcome, we did not observe a higher rate of unfavourable outcomes.

### Overall survival of OHCA patients transported to the hospital

Cox regression analysis of our data revealed that higher age, unwitnessed cardiac arrest, and an initial non-shockable rhythm were independently associated with decreased overall survival in patients with OHCA transferred to a large University hospital, confirming earlier reports (22–25).

A large meta-analysis including 7,397 patients identified younger age, female sex, a shockable rhythm, witnessed arrest, bystander CPR, return of spontaneous circulation and shorter time to cannulation as predictors of increased survival with favourable neurological outcome with ECPR treatment (26).

A recently updated meta-analysis, including 10 observational studies and 3 RCTs, demonstrated a significant reduction in overall mortality among OHCA patients treated with ECPR compared with those receiving conventional CPR (10). However, similar to our results and from the same geographical region, a large French registry study found no significant difference in survival between ECPR and conventional CPR, despite the ECPR cohort having more advantageous baseline characteristics (13).

### Limitations

A limitation of our study is its retrospective, single-centre design, which inherently limits the generalisability of the findings. Neurological outcomes were assessed retrospectively, which may introduce bias. Additionally, the study compares outcomes across different time periods, introducing the possibility that factors unrelated to the implementation of the ECPR programme may have influenced the results. Furthermore, our real-life dataset lacked key time-dependent variables such as exact no and low-flow durations and precise time-to-cannulation, limiting our ability to analyse their influence on outcomes. The temporary suspension of the ECPR programme due to the unprecedented COVID-19 pandemic may have influenced the number of patients treated by ECPR following OHCA, thereby impacting our overall statistics. Similarly, the excess mortality observed in Switzerland during the pandemic in 2020 and 2021 may have affected the results (27). The limited number of patients who received ECPR and survived the event represents another limitation of this study. Finally, given the purely exploratory nature of the results, no adjustment for multiple comparisons was performed.

### Implications and future studies

This study demonstrates the importance of surveillance and outcome data after introducing an intervention (i.e., ECPR programme for patients with refractory cardiac arrest at a Swiss university hospital). Without data, no effect measurements, and no improvements or adaptations to a programme are possible (28). Such data could help provide more evidence for clear criteria to select the patients who would benefit most from an ECPR programme. Future research should aim to identify independent predictors associated with favourable neurological outcome following ECPR, particularly given the considerable variability in prehospital EMS and in-hospital infrastructure across countries and regions.

## Conclusion

This observational study on patients sustaining OHCA transferred to a large Swiss hospital showed that 1-year favourable outcome was 19.7% (95%-CI: 16.6–23.2%). ECPR was provided to 34 patients with refractory OHCA. Of these, all six survivors had a favourable neurological outcome at hospital discharge. No association was found between implementing an ECPR programme for patients with refractory OHCA and 1-year favourable neurological outcome. However, the effect might be underestimated given the low incidence of ECPR.

## Data Availability

The datasets presented in this article are not readily available because the data will be available for researchers with a reasonable research question following approval of the responsible Cantonal Ethics Committee of Bern in collaboration with the authors. Requests to access the datasets should be directed to Alexander Fuchs, alexander.fuchs@insel.ch.
